# Functional, Physicochemical, and Antioxidant Properties of Flour and Cookies from Two Different Banana Varieties (*Musa acuminata* cv. *Pisang awak* and *Musa acuminata* cv. *Red dacca)*

**DOI:** 10.1155/2021/6681687

**Published:** 2021-06-01

**Authors:** Nimesha K. Amarasinghe, Indira Wickramasinghe, Isuru Wijesekara, Gayan Thilakarathna, Sathsara T. Deyalage

**Affiliations:** ^1^Department of Food Science and Technology, Faculty of Applied Sciences, University of Sri Jayewardenepura, Gangodawila, Nugegoda, Sri Lanka; ^2^National Institute of Post-Harvest Management (NIPHM), Anuradhapura, Sri Lanka

## Abstract

Banana *(Musa acuminata)* is grown abundantly in tropical and subtropical countries, and it is consumed as raw or processed. Banana is a significant source of nutrients, and it has been found to contain carbohydrates and other nutritional components. The present study was conducted to evaluate the proximate composition, antioxidant composition, and physicochemical properties of flour obtained from two different banana varieties (*Musa acuminata* cv. *Pisang awak* and *Musa acuminata* cv. *Red dacca)* and to evaluate the proximate composition and antioxidant composition of cookies prepared by incorporating both banana flours. Several sets of cookie samples were prepared separately by incorporating each banana flour where wheat flour and banana flour combinations were 85%-15%, 75%-25%, 70%-30%, and 0-100%. These samples were evaluated for sensory attributes, and two best cookie formulations were selected (70% wheat flour and 30% banana flour) for the analysis. Both *Awak* and *Dacca* had obtained similar amount of carbohydrates *p* < 0.05 while *Dacca* had recorded a higher amount of moisture, fat, protein, ash, and phytonutrients such as polyphenols, antioxidants, and flavonoids. *Dacca* flour had obtained higher values for physicochemical properties like water holding capacity (WHC) and oil holding capacity (OHC). *L*^∗^, *a*^∗^, and *b*^∗^ values were evaluated for banana flour incorporated cookies. Lightness and the redness of cookies were prominent while yellowness was not prominent. There was no significant difference in texture parameters but hardness was higher in banana cookies as they contained a higher content of protein and fiber.

## 1. Introduction

Bananas are known by the scientific name *Musa acuminata* (*Musa* sp.), a very popular fruit in the world market. It can be eaten as raw or processed as well as flour is used as a functional ingredient in various food products [1].

Bananas are cultivated in over 130 countries as the second leading fruit in the world after citrus. As the peel makes up to 35% of the fruit banana peel is rich in dietary fibers, proteins, vitamins and minerals like potassium [2].

Banana is a climacteric fruit, showing an increase in size and carbohydrate deposit in the form of starch. Maturity indices for the harvest of fruits are the age of bunch, cross-sectional angularity, pulp-to-peel ratio, length and diameter of fingers, brittleness of flower end of fruit bunch, firmness of fruit, etc. [3]. The ripening process involves several biochemical pathways such as degradation of starch into sugar, change in the peel and pulp color, and cell wall changes. The maturity stage of bananas is considered a major quality attribute that determines the shelf life of the fruit [4]. Banana pulp is rich in phenolic compounds such as carotenoids, flavonoids, and vitamins (B3, B6, B12, C, and E). Phenolic compounds and natural antioxidants in banana contributes to the storage stability and exerting health benefits as well as contributes to the astringency of green banana [5]. Furthermore, banana is richest with iron and potassium, magnesium, and other nutrient contents.

As highly perishable, the postharvest loss of banana is mainly caused by mechanical, microbiological, and physiological factors. Due to mechanical injuries and abrasion, black color sunken areas can be seen on the skin. Because of that, handlers must be careful to handpick bananas and protect them from damages to the skin [4]. Banana is subjected to fast deterioration due to moisture content and high metabolic activity.

Because of the high perishability, banana needs drying during processing for preserving a longer period. As banana flour is prepared by drying, this flour has a high storability and a long shelf life. Banana flour is prepared, and it is used in bakery and confectionery industries. Banana flour is currently used in the food industry for making bread, cake, pasta, biscuits, cookies, and baby food [5].

Cookies are consumed as snacks because of the crispiness, taste, and digestibility. Most of the time, cookies are prepared using refined wheat flour [6]. Composite flour is healthier because it improves the nutritional value of bakery products when blended with other types of flour. The development of banana flour-incorporated cookies is healthy due to the nutrition content in banana such as carbohydrate, mineral, and antioxidant capacity as well as using banana flour in bakery products also reduced the postharvest loss of banana [7].

The overall objective of this study was to evaluate the nutritional composition (moisture, fat protein, and carbohydrate), physicochemical properties, and antioxidant properties of two selected banana varieties and the development of cookies while reducing the postharvest loss of banana.

## 2. Materials and Methods

### 2.1. Plant Materials

Fully matured but unripened *Musa acuminata* cv. *Pisang awak* and *Musa acuminata* cv*. Red dacca* (3 kg from each) were purchased from a small-scale farmer in Maharagama, Colombo, Sri Lanka.

### 2.2. Chemicals

All chemicals and solvents used were of analytical grade. Methanol, gallic acid, 10% (*v*/*v*) Folin-Ciocalteu reagent, DPPH (2,2-diphenyl-1-picryl-hydrazyl), Trolox (6-hydroxy-2-5-7-8- tetramethylchroman-2-carboxylic acid), sodium acetate trihydrate (C_2_H_3_NaO_2_·3H_2_O), glacial acitic acid (C_2_H_4_O_2_), TPTZ (2,4,6-tri(2- pyridyl)–s-traizine), ferric chloride (FeCl_3_.6H_2_O), HCl, ABTS (2,2-azino-bis(3–ethylbenzthiazoline-6)-sulfonic acid), KH_2_PO_4_ (potassium phosphate dibasic), NaNO_2_, NaCO_3_, H_2_SO_4_, D-glucose, and NaOH, obtained from Sigma Aldrich, St. Louis, MO, USA through Analytical Instrument Pvt, Colombo, Sri Lanka.

### 2.3. Total Soluble Solids

Before the flour preparation, total soluble solids were determined to identify the ripening stage of fruits. For that, 5.0 g of pulp was suspended in 50 mL of water, blended for two minutes, and measured using a refractometer with a scale from 0 to 30° Brix [8].

### 2.4. Banana Flour Preparation

Banana flour was prepared following [9] with some modifications. Raw materials were washed thoroughly with potable water, peeled and sliced (1.5-2 mm thick), and dried at 50°C for 48 h. The dried banana was ground and screened through a 355-micron mesh. Flour was packed in an airtight container at room temperature until analysis.

### 2.5. Physicochemical Properties of Flours

Water holding capacity (WHC) and oil absorption capacity (OAC) were measured to characterize the gel hydration properties of flour. The water holding capacity of banana flour samples was investigated by the modified method described by [10] with modifications. The flour sample, 3.0 g, was dispersed in 25.0 mL of distilled water and placed in a preweighed centrifuge tube. The dispersion was vigorously mixed for 1 min and was left at room temperature for 1 h and centrifuged at 3000 × g for 25 min. The supernatant was discarded, and the tube was weighed. The water holding capacity according to the weight of samples was calculated, and results are expressed as gram of water retained per gram of flour (DW).

The determination of the oil holding capacity of flour samples was followed according to the modified method described by [11]. The flour sample (1.0 g) was measured to a preweighed centrifuge tube, and 12.0 mL coconut oil was added and mixed using the vortex mixer. Tubewas allowed to stand at ambient temperature (30 ± 2°C) for 1 h with agitation and centrifuged for 25 min at 3000 × g, and the oil supernatant was removed and measured. The OHC of flour samples was expressed as the number of grams of oil held by 1.0 g of sample (DW).

### 2.6. Development of Banana Flour-Incorporated Cookies

Cookies were prepared using each banana flour combining with wheat flour according to the method proposed by ([12, 13] with slight modifications.

To test the suitability of banana flour in cookies, the cookies were standardized by blending with wheat flour in varying wheat flour: banana flour ratios *T*_1_—85 : 15, *T*_2_—80 : 20, *T*_3_—70 : 30, and *T*_0_—100 : 0 for *Awak* and *T*_1_^−^—85 : 15, *T*_2_^−^—80 : 20, *T*_3_^−^—70 : 30, and *T*_0_^−^—100 : 0 for *Dacca.*

Control formulations (*T*_0_, *T*_0_^−^) comprised of 100 g wheat flour, margarine (45.0 g), sugar (30.0 g), egg (31.0 g), powdered milk (8.0 g), salt (1.0 g), vanilla (2.0 mL), and sodium bicarbonate (1.0 g). Sugar was ground to a fine powder and mixed with margarine for 5 min to make the cream. Eggs and powdered milk were added while mixing. l Four, salt, and vanilla flavor and sodium bicarbonate were thoroughly mixed and added to the cream mixture to form the dough. The dough was covered with a polythene and kept in the freezer for 30 min to rest. It was kneaded to a uniform thickness (5 mm) and cut into a uniform diameter (6 mm) using a cutter then kept on a tray. The tray was kept in the oven and baked at 180°C for 20 min. Baked cookies were cooled at ambient temperature, packed in high-density polythene, and labelled and stored at ambient temperature.

### 2.7. Sensory Analysis

Cookie samples were prepared using both flour varieties and coded with different three-digit random numbers, and the randomized order of the samples was presented to each panellist. Two cookie varieties were tested separately.

Sensory quality attributes (appearance, texture, odor, taste, aftertaste, and overall acceptability) were evaluated by a sensory panel of 30 semitrained members using a 5-point Hedonic scale. After carrying out the sensory evaluation, the best combinations of *Awak* and *Dacca* flour cookies were selected and used in the analyses [14].

### 2.8. Proximate Composition Analysis of Flour and Cookies

The chemical composition such as moisture, fat, protein content, and ash content of samples was determined as outlined in AOAC methods (925.10, 922.06, 920.87, and 923.03, respectively) [15]. Carbohydrate content was determined by following the below equation:

(1) $$\begin{array}{c} \text{Carbohydrate}=100\%-\left(\text{moisture}-\text{protein}-\text{fat}-\text{ash}\right)\%. \end{array}$$

Results were recorded on dry basis (DW).

### 2.9. Analysis of Phytochemical Contents

#### 2.9.1. Sample Extraction

The extraction was carried out according to the procedure outlined in [16] with some modifications. Approximately 2.5 g of each powder sample was extracted with 25.0 mL of 70% methanol using a mechanical shaker (150 rpm) for an overnight at room temperature. The extract was centrifuged (HERMLE, Z326K) for 15 min at 3000 rpm and filtered through Whatman No. 01 filter paper to obtain a particle-free extract. The extracted solutions were kept at 4°C for subsequent analysis.

#### 2.9.2. Total Phenolic Content (TPC)

Determination of total phenolic content was conducted according to ISO/DIS 14502-1. Folin-Ciocalteu reagent, 2.5 mL, was added to 0.5 mL sample extract, and the content was mixed thoroughly. Within 3–8 min, 2.0 mL of Na_2_CO_3_ (7.5%) was added into each tube and mixed well. Tubes were allowed to stand at room temperature for 60 min, and then, the absorbance value of each sample was measured at 765 nm using UV-Vis Spectrophotometer (HATCH DR 600). A control sample was prepared by mixing 0.5 mL of 70% methanol with 2.5 mL of Folin- Ciocalteu reagent and 2.0 mL of Na_2_CO_3_. The standard curve was evaluated from regression analysis (*y* = 0.0122*x*–0.054, *R*^2^ = 0.9972). The results were expressed as gallic acid equivalent (GAE) in milligram per g of plant material (DW).

#### 2.9.3. DPPH Radical Scavenging Activity

DPPH radical scavenging activity was assessed by using the method suggested by [17] with some modifications. A control sample was prepared by mixing 2.0 mL DPPH solution in 2.0 mL methanol to obtain an absorbance of 0.900 ± 0.02 units at 517 nm using UV-Vis Spectrophotometer. Initially, 2.0 mL of 0.16 mM DPPH methanolic solution was added to 2.0 mL methanolic sample and vortexed (VELP SCIENTIFICA ZX3) well. Samples were kept in dark for 30 min. The absorbance was read at 517 nm using a UV-Vis spectrophotometer. The results were expressed as Trolox equivalent (TE) in milligram per g of dried sample using a calibration curve of Trolox (*y* = −0.0464*x* + 0.8384, *R*^2^ = 0.9837).

#### 2.9.4. FRAP Assay (Ferric Reducing Antioxidant Power)

Ferric reducing antioxidant power levels were determined using a method proposed by [18] with some modifications. FRAP reagent was prepared by mixing 300 mM acetate buffer, pH 3.6 (3.1 g sodium acetate trihydrate (C_2_H_3_NaO_2_·3H_2_O) with 16.0 mL glacial acetic acid (made up to 1 liter with distilled water), 10 mM TPTZ in 40 mM HCl, and 20 mM ferric chloride in the ratio of 10 : 1 : 1 ratio to give the working FRAP reagent.

A sample extract, 0.15 mL, was introduced into a tube, and 4.50 mL of FRAP reagent was added. The mixture was vortexed well, and the tube was incubated at 37°C for 30 min. The absorbance of the mixture was measured at 593 nm using a UV-Vis spectrophotometer. The results were expressed as Trolox equivalent (TE) in milligram per g of dried sample using a calibration curve of Trolox (*y* = 0.0072*x* + 0.0332, *R*^2^ = 0.9938).

#### 2.9.5. ABTS Assay

ABTS radical scavenging activity was determined according to the method employed by [19] [20] with slight modifications. Previously prepared ABTS solution and potassium persulfate **(**K_2_S_2_O_8_) were mixed in 1 : 1 ratio. The reaction mixture was left to stand at 37°C for 16 h under dark conditions. ABTS/K_2_S_2_O_8_ solution was diluted with 70% methanol with a ratio of 1 : 40 (to obtain an absorbance at 0.8000-0.700). 0.04 mL of the sample extract was added to 4.0 mL of ABTS/K_2_S_2_O_8_ solution. The solution was mixed well and kept in dark for 15 min. The absorbance of the samples was measured at 734 nm using a UV-Vis spectrophotometer. The results were expressed as Trolox equivalent (TE) in milligram per g of dried sample using a calibration curve of Trolox (*y* = −0.0012*x* + 0.756, *R*^2^ = 0.9946).

#### 2.9.6. Total Flavonoid Content (TFC)

The total tlavonoid content was determined by the method as outlined in [21] with slight modifications. Approximately 2.50 mL aliquot, 150.0 *μ*L of NaNO_2_ (5%), 150.0 *μ*L of AlCl_3_ (10%), and 1.0 mL of sodium hydroxide (NaOH) (1 M) were pipetted into tubes, respectively. After that, the solution volume was increased up to 5.0 mL by adding distilled water. The content of the mixture was vortexed properly, and the absorbance of each sample was measured at 510 nm using a UV-Vis spectrophotometer. The results were expressed as quercetin equivalent (QE) in milligram per g of the dried sample using a calibration curve of quercetin (*y* = 0.0019*x* + 0.0436, *R*^2^ = 0.9947).

### 2.10. Chromameter Value Comparison for Cookies

The color of cookies was evaluated according to *L*^∗^ (lightness (*L* = 100; white and *L* = 0; black)), *a*^∗^ (green (−60) to red (+60)), and *b*^∗^ (blue (−60) to yellow (+60)). *L*^∗^, *a*∗, and *b*^∗^ color values for cookies were measured by using chromameter (CR-400). Color values were taken as replicate (*n* = 3) in different areas of the biscuit surface [14].

### 2.11. Instrumental Texture Analysis of Cookies

The texture profile analyses (TPA) for banana flour cookies were carried out using a Brookfield CT3 Texture Analyzer coupled to Brookfield Texture Pro CT software. Tests were performed according to the parameters described in [22]. Cookies made out of 100% wheat flour were used as the control sample. Compression test was performed on cookies using 2.0 mm cylindrical, 5.0 g stainless steel, 20.0 mm probe (TA39), load of 10.0 g trigger, and test speed 1.0 mm/s. Two successive compressions were carried out on each sample. The resulting force-time curves were developed for hardness, chewiness, gumminess, springiness, and cohesiveness. The same textural properties were measured for randomly selected cookies from each variety. All results were expressed in a report with values automatically calculated by the analyzer's software.

#### 2.11.1. Statistical Analysis

The study was performed in triplicate for each sample, and data obtained from the study were expressed as mean ± standard error (SE) and statistical analysis performed by using the statistical software MINITAB R 17. Oneway ANOVA and Turkey's mean separation tests were carried out to evaluate the proximate composition and antioxidant capacity at the significant level 0.05. Regression analysis was conducted when constructing standard curves in TPC, DPPH, FRAP, ABTS, and TFC assays. One sample two test was used in analyzing the physicochemical properties of flour. Sensory data analyzed by the nonparametric Friedman test at 95% confidence level by using statistical software SPSS21.

## 3. Discussion

### 3.1. Total Soluble Solids

Brix values of bananas are shown in Table 1. As fully matured but unripened bananas were used in the experiment, the Brix value was in the range 14.17–15.27. During ripening, the total sugar in the fruit is progressively increased while starch content is decreased due to the hydrolysis of starch into sugar. At the maximum maturity stage, the maximum TSS had recorded as (23.07° Brix) according to [23]. Increment in TSS is an important trait of hydrolysis of starch into soluble sugars such as glucose, sucrose, and fructose. [23], and the trend of increasing Brix values indicates higher maturity stages had high sugar content.

### 3.2. Physicochemical Properties

Hydration properties of different banana flours are shown in Table 2. WHO of *Awak* and *Dacca* ranged from 2.43 to 2.90 while OHC ranged from 1.16 to 1.45, respectively. In the presence of water, hydrogen bonds are formed with the hydroxyl groups of starch. Reduction of hydration properties is an indication of starch degradation and sugar release because sugar could interact with starch chains and limit the availability of water to hydrate the starch. There was a significant (*p* < 0.05) decrease in WHC in *Awak* (2.43) as they contain more sugars and reduce the hydration properties by limiting the availability of water to hydrate the starch [24]. According to [25], WHC of the banana peel flour was recorded as 4.29*c* ± 0.17 while wheat flour has recorded WHC as 2.12^*a*^ ± 0.11.

OHC indicates the emulsifier role of fat and it was increased in *Dacca* (1.45) but there was no significant difference in OHC between *Dacca* and *Awak* (*p* > 0.05). The oil holding capacity (OHC) of flour is equally important as it improves the mouthfeel and retains the flavor [26]. According to [25], OHC of banana peel flour has been recorded as 2.01*c* ± 0.37 while OHC of wheat flour has been recorded as 1.15*a* ± 0.49.

### 3.3. Nutritional and Chemical Properties of Flour and Cookies

Table 3 summarizes the chemical composition of banana flour and cookies. Results indicate that the variety of banana significantly influenced the chemical composition (*p* = 0.000) of the flour and cookie. There was a significant difference (*p* < 0.05) in the proximate composition, TPC, antioxidant content, and TFC of the banana flour and cookies. Both proximate and phytochemical contents were higher in banana flour compared to cookies except protein and fat as they were added externally in cookie preparation.

The moisture content of flour obtained from *Awak* had the lowest value (8.00%), while the *Dacca* flour had recorded the significantly (*p* < 0.05) largest one (8.84%). These values were within the acceptable limits (<20.0%) to reach a stable shelf life. According to [25], if the flours' moisture content is less than 14%, it can resist microbial growth and contribute to storage. The moisture content of *Dacca* flour-incorporated cookies was higher (3.79%). Higher water binding capacity in *Dacca* flour was attributed to the high water binding capacity in the cookies [7]. According to [27], moisture content of wheat-guava peel biscuits ranged from 2.7 to 4.9%.

Protein is a main requirement for growth, repair, and maintenance of the human body as well as to maintain fluids and protein acts as enzymes, hormones, etc. Protein content was also significantly high (*p* < 0.05) in *Dacca* flour (5.88%) compared to *Awak* (4.81%). Both cookies had recorded a significantly higher protein content compared to relevant flour as eggs were added in the preparation of cookies and egg is known as a good protein source [7] The result of the protein content of *Awak*-*Dacca* flour-incorporated cookies ranged from 9.22% to 10.73% where there was a significant difference. According to [28], the protein content of 20 : 80 (banana flour : wheat flour) biscuit formulation was recorded as 7.89%.

Fat contents were slightly low in both flour types. The result of the fat content of *Awak*-*Dacca* ranged from 1.24% to 2.16% where there was a significant difference. As the same quantity of fat added, the fat content of both cookies has not differed much. Moreover, the results for the fat content of cookies ranged from 15.00% to 15.60%. According to [29], the fat content of soft dough biscuits should be within the standard value 15%-20%. According to [28], the fat content of cookies prepared from composite flours should contain a fat content of 15.1%-18.1%. The fat content of prepared cookies was within the standard range [30]. According to [28], protein content of 20 : 80 (banana flour : wheat flour) biscuit formulation was recorded as 17.21%.

Amylases in the unripe fruit transform starch into sugars during maturation. Starch is mainly converted into several sugars like sucrose, glucose, fructose, and small quantities of maltose and rhamnose as the total soluble solid contents in banana increase with fruit ripening [5]. Apart from starch and sugar, both banana pulps contain some nonstarch polysaccharides, including cellulose like insoluble fiber and pectin and hemicelluloses like soluble fiber [9]. According to [31], the principle component of unripen banana flour is starch (63.50%-74.65%).

*Awak* flour had recorded the highest carbohydrate content (77.14%) because *Awak* contains more sugar than *Dacca* flour (74.28%) but the contents were not significantly different (*p* > 0.05). The carbohydrate content of cookies was significantly different (*p* > 0.05). Moreover, the results for carbohydrate content of *Dacca-Awak* flour-incorporated cookies ranged from 66.21% to 68.96%. According to [28], the carbohydrate content of 20 : 80 (banana flour : wheat flour) biscuit formulation was recorded as 71.64%.

As sugar and wheat flour are added in cookie preparation, the carbohydrate content in cookies should be higher than flour. But in the baking process, glucose and fructose had degraded in Maillard reaction and caramelization. Each of these reactions had decreased the carbohydrate content in cookies compared to flour [32].

Ash content represents all the inorganic minerals that consists the sample. When comparing flour and cookies, flour had recorded a higher amount of ash. The results for ash content of *Awak-Dacca* ranged from 3.03% to 3.25%. The overall mineral content of *Awak* flour was relatively lower. Moreover, the results for ash content of *Awak-Dacca* flour-incorporated cookies ranged from 1.62% to 1.87%. Moreover, these results were similar as outlined in [31] (2.02%). According to [33], a relative concentration of minerals found in banana *Awak* flour while the highest mineral content was found in potassium (982 mg/100 g dry matter), followed by magnesium, calcium, sodium, and other minerals. Moreover, *Awak* contained lower mineral content when compared with other banana cultivars. According to [28], the ash content of 20 : 80 (banana flour : wheat flour) cookie formulation has been recorded as 1.17%.

Phenolic compounds are considered primary antioxidants or free radical terminators which have the ability to donate hydrogen atoms to free radicals. But at certain times, antinutrients reduce the nutritional values of food by reducing the bioavailability, digestibility, and utilization of nutrients. Banana contains various phenolic compounds, such as gallic acid, catechin, epicatechin, tannins, anthocyanins, catecholamines, phenolic acids, and flavonoids [1]. Availability and quantity of these nutrients in the banana are influenced by factors like ripening stages of the fruit, location, climatic factor, and agricultural and cultural practice [25].

TPC of *Awak-Dacca* flour ranged from 3.58 to 4.95 mg GE/g, and there was a significant difference (*p* < 0.05). These results are aligned with results recorded by [34]. Moreover, TPC of banana flour in this study was lower than the TPC of guava peel flour (8.87) [27]. Furthermore, TPC of *Awak* and *Dacca* cookies was 1.10 and 3.99 mg GE/g, respectively. TPC of cookies had reduced due to the sensitivity of vitamin C (ascorbic acid) to heat. In baking polymerization of polyphenols, decarboxylation of phenolic acids and Maillard-like reactions occurred due to heat and the TPC content is drastically decreased [25]. [35] expressed that TPC decreased as free phenolics reduced and bound phenolics increased. At high temperatures, phenolic compounds lead to reduced chemical activity or used in polymerization.

Banana being abundant in antioxidants has contained bioactive compounds such as phenolics, carotenoids, dopamine, dopa, carotenes, norepinephrine, and ascorbic acid (vitamin C) which have the ability to protect the body against oxidative stress. [36] expressed that the antioxidant compound gallocatechin was abundant in banana.

DPPH ranged from 0.15 to 0.68 mg GE/g in *Awak-Dacca* flour, respectively, while significantly different (*p* < 0.05). Effect of extracting solvent which dissolved the sample has contributed to the behavior of the obtained results of flours. DPPH increases when the concentration of phenolic compounds and their hydroxylation increase. The DPPH ranged from 0.04 to 0.15 mg TE/g in *Awak-Dacca* cookies. The highest DPPH was obtained in *Dacca* flour and *Dacca* cookies. [25] stated that baking and microwave roasting increase the antioxidant activity of baked products as well as during baking antioxidant capacity become low as in baking due to polymerization like reactions.

FRAP values ranged from 25.35 to 28.75 mg TE/g in *Awak* and *Dacca* flours, respectively, and there was a significant difference (*p* < 0.05). FRAP values of the cookies ranged from 3.39 to 7.32 mg TE/g in *Awak* and *Dacca* cookies. *Dacca* still had significantly higher (*p* < 0.05) antioxidant activity than *Awak*. ABTS values of *Awak* and *Dacca* flours ranged from 59.65 to 68.06 mg TE/g, and results for *Awak* and *Dacca* flour cookies were 37.92-56.80 mg TE/g. Both *Awak* flour and *Awak* cookies had recorded the lower values for ABTS. The incorporation of banana flour into wheat flour significantly improved the antioxidant capacity of composite flours compared to wheat flour.

According to [25], the FRAP value of banana flour was recorded as 1.41^*d*^ ± 0.01 while the FRAP value of 4P% banana flour : 96% wheat flour-incorporated cookies was recorded as 0.64^*b*^ ± 0.01. According to (adedayo), the ABTS value of Cavendish banana 5.03 ± 0.08^a^ mmol TEAC/g was recorded and *Red dacca* was recorded as 4.98 ± 0.11^a^ mmol TEAC/g.

Banana flour is high in TFC compared to wheat flour, and the main classes of flavonoids detected in bananas are quercetin, myricetin, and kaempferol. According to [37], flavonoids are active against many infectious diseases (bacterial and viral diseases), cardiovascular diseases, cancers, and other age-related diseases. Flour had obtained lower TFC values while cookies had recorded higher TFC values than flours. *Awak* and *Dacca* flours ranged from 0.20 to 0.27 mg QE/g while *Awak* cookies and *Dacca* cookies ranged from 0.45 to 0.68 mg QE/g. Baking contributed to a significant increase (*p* < 0.05) in TFC of cookies compared with the flours.

According to [25], the results of TFC of wheat flour and banana flour and ranged from 11.69^b^ ± 0.05 to 23.19^e^ ± 0.11 and TFC of 4% banana flour : 96% wheat flour-incorporated cookies was 33.74^d^ ± 0.17. Baking contributed to a significant increase in TFC of cookies compared to banana flour. This difference can occur due to the development of meladoins. Meladoins, brown color pigments, are the products of Maillard browning reaction which occur during the baking process. Meladoins are produced as a result of high temperatures due to reactions between amino acids and reducing sugars, and they give different flavors and brown color in baked food. Based on the literature [38], formulation of Maillard reaction products/meladoins can be described in three main stages. The first stage is known as Amadori rearrangement, and 1-amino-1deoxy2 ketose is formed due to the reaction between sugars and amino acids. In the second stage, sugar molecules are subjected to dehydration and fragmentation as well as amino acid are also degraded. In this intermediate stage, hydroxymethylfurfural (HMF) fission products such as pyruvaldehyde and diacetyl are formed. As the final stage, aldol condensation occurs and highly colored meladoins (heterocyclic nitrogenous compounds) are formed.

### 3.4. Sensory Evaluation of Cookies

Mean sensory scores for sensory attributes of the cookies produced by wheat-banana flour and banana flour blends at different proportions are shown in Table 4. Two separate sensory evaluation tests were carried out to select the best formulation for *Awak* flour-incorporated cookies (treatment *T*_1_, *T*_2_, *T*_3_, and *T*_0_) and *Dacca* flour-incorporated cookies (treatment *T*_1_^−^, *T*_2_^−^, *T*_3_^−^, and *T*_0_^−^). The sensory scores for the sensory attributes of cookie samples varied significantly (*p* < 0.05).

Cookie, treatment *T*_3_ (70 : 30) was better accepted by panel members as the best *Awak* flour-incorporated cookie. However, *T*_3_ was compared favorably well with treatments *T*_1_, *T*_2_, and *T*_0_ (control) in texture, appearance, odor, taste, aftertaste, and overall acceptability. Treatment *T*_2_ had the highest rating for appearance. The lower ratings for appearance in treatment *T*_3_ may be due to the initial dark color in banana flour compared to the wheat flour that is lighter in color. The color or appearance of cookies becomes darker in the baking process as the chemical reactions take place due to higher sugar content. Treatments *T*_1_, *T*_2_, and *T*_0_ (control) had almost similar scores for overall acceptability while sample *T*_3_ has recorded the highest.

Treatment *T*_3_^−^ (70 : 30) was better accepted by panel members as the best *Dacca* flour-incorporated cookie from the sensory evaluation results Table 4 as treatment *T*_3_^−^ has obtained the higher mean score for all the attributes. Treatment *T*_0_^−^ (control) had the lowest scores for all the attributes. Treatments *T*_1_^−^ and *T*_2_^−^ had obtained almost similar scores. Treatment *T*_3_^−^ has recorded the highest score for appearance due to low sugar content (less darkness) [30]. Considering the sensory evaluation test results, the best two formulations were selected and used in chemical composition analyses.

A similar study has been done to evaluate the sensory attributes of cookies prepared from wheat flour and pitaya (*Hylocereus undatus*) peel flour blends. According to [14], the value of the color attribute of 100% wheat flour cookies was 5.73^b^ ± 1.31 while the value of 15% pitiya peel flour : 75% wheat flour cookies was 4.93^ab^ ± 1.48. The value for overall acceptability of 100% wheat flour cookies was 4.97^a^ ± 1.45, and the value for overall acceptability of 15% pitaya flour : 75% wheat flour cookies was 5.23^a^ ± 1.54, respectively.

### 3.5. Color Attribute of Cookies

Table 5 shows the color attributes of cookies. The color of cookies was represented in terms of lightness (*L*^∗^), redness (*a*^∗^), and yellowness (*b*^∗^).

The *L*^∗^, *a*^∗^, and *b*^∗^ values of *Awak* and *Dacca* flour-incorporated biscuits ranged from 59.18 to 60.29, 6.82 to 9.20, and 28.94 to 31.12, respectively. The color changes occurred due to the extent of Maillard reaction as banana contains glucose, fructose, and protein. The least *L*^∗^ value was observed in *Awak* flour-incorporated cookies because caramelization or Maillard reaction caused browning during baking of banana flour at high temperature as *Awak* contains more sugar. As *a*^∗^ values indicate the redness of cookies both banana flour-incorporated cookies had obtained positive *a*^∗^ values which were significantly different (*p* < 0.05). The redness of the crust was higher in *Dacca* flour-incorporated cookies due to the highest antioxidant and phenolic content. The *b*^∗^ color value represents the yellowness of biscuits, and the yellowness of both biscuits was more prominent than redness [14]. According to [25], *L*^∗^, *a*^∗^, and *b*^∗^ values of 100% wheat flour-incorporated biscuits were recorded as 66.00, 4.06, and 27.53, respectively.

According to [25], 4% banana peel flour : 96% wheat flour-incorporated cookies has recorded *L*^∗^, *a*^∗^, and *b*^∗^ values as 51.767^c^ ± 0.945, 5.433^b^ ± 0.473, and 20.467^c^ ± 0.378, respectively, while 100% wheat flour cookies has recorded *L*^∗^, *a*^∗^, and *b*^∗^ values as 65.000^d^ ± 0.529, 4.067^a^ ± 0.058, and 27.533^d^ ± 0.757. *L*^∗^, *a*^∗^, and *b*^∗^ values of cookies have been decreased with the substitution of banana peel flour.

### 3.6. Textural Properties of Cookies

Textural quality is one of the most important quality attributes for cookies. The texture of the two best banana cookie formulations was tested against the texture of 100% wheat flour cookie which was considered a commercial type cookie. The primary TPA parameters, hardness, springiness, and cohesiveness, and secondary parameters of gumminess and chewiness are shown in Table 6.

There were no significant (*p* < 0.05) differences in the texture parameters of the cookies. Hardness can be defined as the peak force required to break the cookie, increased as incorporating banana flour. Baking conditions, the type and quantity of ingredients, and protein content and fiber content of the flour influence its hardness and other textural attributes. The mechanical changes attributed to the replacement of wheat flour by banana flour in cookies are related to the increases of fibers, liquid oil, proteins, and reduction in carbohydrates. Cookies are made out of a continuous glass-like sugar structure in which ungelatinized starch granules and fat are embedded in the structure. Due to starch gelatinization recrystallization, protein denaturation has occurred and the structure and texture are developed. Though protein content is higher in cookies, the gluten content is reduced in the dough. And also, in baking, moisture migrates from the wet area to the drier surface and the product is turning in dry.

There was no significant difference in texture parameters but hardness is higher in banana cookies as they contained a higher content of protein and fiber. Protein and fiber are more important for texture than the lowering of carbohydrates (starch). Previous studies [39] also discovered the positive relation of dietary fibers and hardness and gumminess [22].

According to [25], hardness of 100% wheat flour cookies was recorded as 2245.38^a^ ± 1.17 while the hardness of 4% banana peel flour : 96% wheat flour-incorporated cookies was recorded as 3572.83^d^ ± 1.14.

According to [40], 100% wheat flour cookies and 30% okara flour : 70% wheat flour cookies have recorded hardness values of 22.08 ± 3.5^a^ and 31.01 ± 6.0^c,b^. It states that increasing of okara flour is affected by the fracture force and the fracture force is increased as the okara flour content is increased. Because the dough structure is compacted by proteins and fiber.

## 4. Conclusion

The results clearly revealed the proximate composition and antioxidant composition of banana flour (*Pisang awak* and *Red dacca*) were higher than banana cookies except for protein and fat as they are added in the cookie preparation process. OHC in *Red dacca* was higher and WHC in *Pisang awak* was less than *Red dacca* as they contain more sugars and reduce the hydration properties by limiting the availability of water to hydrate the starch. Cookies enriched with banana flour reduce the postharvest loss of banana while improving the nutritional quality of cookies. Textural attributes of cookies are increased with the incorporation of banana flour instead of using 100% wheat flour in cookies. *L*^∗^, *a*^∗^, and *b*^∗^ values of cookies have been decreased with the substitution of banana flour. Among the three formulations, 70% wheat flour : 30% banana flour cookies achieved the highest value for the overall performance.

## Figures and Tables

**Table 1:** Brix values of *Pisang awak* and *Red dacca.*

Sample	Brix
*Pisang awak*	15.27 ± 0.25
*Red dacca*	14.17 ± 0.29

**Table 2:** Physicochemical properties of flour and cookies.

Parameter	*Pisang awak*	*Red dacca*
WHC (g water/g flour)	2.43 ± 0.02^b^	2.90 ± 0.03^a^
OHC (g water/g flour)	1.16 ± 0.09^b^	1.45 ± 0.12^a^

Values are presented as mean ± sd, *n* = 3; values in the same row having the same superscript letters are not significantly different (*p* > 0.05).

**Table 3:** Proximate composition of the flour and cookies.

Parameter	*Pisang awak* flour	*Red dacca* flour	*Pisang awak* flour-incorporated cookies	*Red dacca* flour-incorporated cookies
Moisture (%)	8.00 ± 0.15^b^	8.84 ± 0.43^a^	3.62 ± 0.03^c^	3.79 ± 0.05^c^
Protein (%)	4.81 ± 0.31^d^	5.88 ± 0.19^c^	9.22 ± 0.187^b^	10.73 ± 0.26^a^
Fat (%)	1.24 ± 0.05^d^	2.16 ± 0.14^c^	15.00 ± 0.15^b^	15.60 ± 0.41^a^
Ash (%)	3.03 ± 0.21^a^	3.25 ± 0.31^a^	1.62 ± 0.09^b^	1.87 ± 0.06^b^
Carbohydrate (%)	77.14 ± 0.46^a^	74.28 ± 0.37^a^	68.96 ± 0.44^c^	66.21 ± 0.39^d^
TPC (mg GAE/g)	3.58 ± 0.10	4.95 ± 0.49	1.10 ± 0.08	3.99 ± 0.07^b^
DPPH (mgTE/g)	0.15 ± 0.02^b^	0.68 ± 0.10^a^	0.04 ± 0.01^b^	0.15 ± 0.02^a^
FRAP (mg TE/g)	25.35 ± 1.34*b*^b^	28.75 ± 1.02^a^	3.39 ± 0.30^d^	7.32 ± 0.57^c^
ABTS (mg TE/g)	59.65 ± 5.02^a^	68.06 ± 1.20^a^	37.92 ± 3.25^c^	56.80 ± 0.64^b^
TFC (mg QE/g)	0.20 ± 0.01^d^	0.27 ± 0.01^b^	0.45 ± 0.004^c^	0.68 ± 0.001^a^

Values are presented as mean ± sd, *n* = 3. Values in the same row having the same superscript letters are not significantly different (*p* > 0.05).

**Table 4:** Friedman test values for sensory attributes of *Pisang awak* and *Red dacca* flour-incorporated cookies.

Treatment number	Appearance	Texture	Taste	Odor	Aftertaste	Overall acceptability
*T*_1_	2.07	2.67	2.57	2.67	2.73	2.42
*T*_2_	3.22	2.17	1.97	2.15	2.27	2.27
*T*_3_	2.62	3.05	3.57	3.07	3.27	3.48
*T*_0_	2.10	2.12	1.90	2.12	1.73	1.83
*T*_1_^−^	2.38	2.35	2.47	2.18	2.62	2.52
*T*_2_^−^	2.15	2.70	2.62	2.73	2.57	2.40
*T*_3_^−^	3.55	3.07	3.50	3.00	3.27	3.42
*T*_0_^−^	1.92	1.88	1.42	2.08	1.55	1.67
*p* value	0.000	0.003	0.000	0.003	0.000	0.000

**Table 5:** Color attribute of cookies.

Type of biscuit	*L* ^∗^	*a* ^∗^	*b* ^∗^
Pisang awak	59.18 ± 3.46^a^	6.82 ± 0.37^b^	28.94 ± 1.64^b^
Red dacca	60.29 ± 2.28^a^	9.20 ± 1.06^a^	31.12 ± 1.64^a^

Values are presented as mean ± sd, *n* = 3. Values in the same row having the same superscript letters are not significantly different (*p* > 0.05).

**Table 6:** Textural properties of cookies.

Parameter	*Pisang awak* flour-incorporated cookies	*Red dacca* flour-incorporated cookies	Wheat flour cookies
Hardness(g)	3374 ± 421^a^	3082 ± 744^a^	3005 ± 723^a^
Springiness(mm)	1.04 ± 0.83^a^	4.51 ± 5.96^a^	0.85 ± 0.52^a^
Gumminess(g)	674 ± 785^a^	771 ± 804^a^	458 ± 251^a^
Cohesiveness	0.19 ± 0.20^a^	0.27 ± 0.29^a^	0.12 ± 0.05^a^
Chewiness (mJ)	10.68 ± 12.76^a^	22.28 ± 19.82^a^	4.82 ± 5.94^a^

Values are presented as mean ± sd, *n* = 3. Values in the same row having the same superscript letters are not significantly different (*p* > 0.05).

## Data Availability

All the data relevant to the research can be found in the manuscript. Any further information is available upon the request from the author.
